# Distinct temporal recruitment of *Plasmodium* alveolins to the subpellicular network

**DOI:** 10.1007/s00436-014-4093-4

**Published:** 2014-09-04

**Authors:** Annie Z. Tremp, Fatimah S. Al-Khattaf, Johannes T. Dessens

**Affiliations:** 1 Pathogen Molecular Biology Department, Faculty of Infectious and Tropical Diseases, London School of Hygiene and Tropical Medicine, Keppel Street, London, WC1E 7HT UK; 2 Department of Infection Control, College of Medicine, King Saud University, Riyadh, Saudi Arabia

**Keywords:** *Plasmodium berghei*, Cytoskeleton, Intermediate filament, Sexual stages, Sporogonic development

## Abstract

The zoite stages of malaria parasites (merozoite, ookinete and sporozoite) possess a distinctive cortical structure termed the pellicle, which is defined by a double membrane layer named the inner membrane complex (IMC). The IMC is supported by a cytoskeleton of intermediate filaments, termed the subpellicular network (SPN). *Plasmodium* IMC1 proteins, or alveolins, make up a conserved family of structurally related proteins that comprise building blocks of the SPN. Here, using green fluorescent protein (GFP) tagging in *P. berghei*, we show that the alveolins *Pb*IMC1c and *Pb*IMC1e are expressed in all three zoite stages. Our data reveal that *Pb*IMC1e is assembled into the SPN concurrent with pellicle development, while *Pb*IMC1c is assembled after pellicle formation. In the sexual stages, these processes are accompanied by different gene expressions from maternal and paternal alleles: *Pb*IMC1e is expressed uniquely from the maternal allele, while *Pb*IMC1c is expressed from the maternal allele in gametocytes, but from both parental alleles during ookinete development. These findings establish biogenesis of the cortical cytoskeleton in *Plasmodium* to be a complex and dynamic process, involving distinct parental gene expression and chronological recruitment of its protein constituents. While allelic replacement of the *pbimc1c* and *pbimc1e* genes with GFP-tagged versions was readily achieved using double crossover homologous recombination, attempts to disrupt these genes by this strategy only resulted in the integration of the selectable marker and GFP reporter into non-specific genomic locations. The recurrent inability to disrupt these genes provides the first genetic evidence that alveolins are necessary for asexual blood-stage parasite development in *Plasmodium*.

## Introduction

Malaria parasite transmission is initiated by the ingestion of gametocytemic blood by a vector mosquito, which initiates gametogenesis followed by fertilization. Zygotes transform into motile ookinetes that traverse the gut wall of the insect and transform into oocysts (Meis & Ponnudurai, 1987; Meis et al., 1989). An approximately 2-week period of growth and replication culminates in hundreds of motile sporozoites being released from each oocyst. These invade the salivary glands and are transmitted to new hosts, again by blood feeding of the insect. Once in the host, sporozoites rapidly infect liver cells and replicate each to produce thousands of merozoites. The motile merozoites are released into the bloodstream, where they infect red blood cells and either replicate to form more merozoites or differentiate into sexual-stage male and female gametocytes to complete the life cycle.

The three motile and invasive stages (zoites) of *Plasmodium* species (i.e. ookinetes, sporozoites and merozoites), as well as zoites of other apicomplexan parasites, possess a similar cortical structure termed the pellicle. The pellicle is essentially made up of the plasma membrane and an underlying double membrane structure termed the inner membrane complex (IMC) (Bannister et al., 2000; Morrissette & Sibley, 2002; Santos et al., 2009). Closely associated with the IMC on its cytoplasmic side is a network of intermediate filaments termed the subpellicular network (SPN), which supports the pellicular membranes and provides mechanical strength to the cell (Mann & Beckers, 2001). The pellicular membranes are further supported by subpellicular microtubules that run lengthwise from the anterior towards the posterior end, completing the cortical cytoskeleton (Bannister et al., 2000; Morrissette & Sibley, 2002; Santos et al., 2009).

Several members of an Apicomplexa-specific family of proteins termed IMC1 proteins have been identified as components of the SPN (Khater et al., 2004; Mann & Beckers, 2001). Structurally related proteins from ciliates and dinoflagellate algae have since been added to this protein family renamed ‘alveolins’, which now defines the Alveolata infrakingdom (Gould et al., 2008). In the genus *Plasmodium*, the number of members of the alveolin family has risen to 12 (Kono et al., 2012), which are encoded by conserved and syntenic genes. The alveolin family members display differential expression between the three zoite stages of the parasite, with the largest repertoires present in the ookinete and sporozoite according to proteomic studies (Florens et al., 2002; Hall et al., 2005; Lasonder et al., 2002; Lindner et al., 2013; Treeck et al., 2011). It has been shown in the rodent malaria species *Plasmodium berghei* that the disruption of individual alveolin family members expressed in sporozoites (*Pb*IMC1a), in ookinetes (*Pb*IMC1b) or in both these zoites (*Pb*IMC1h) results in morphological abnormalities that are accompanied by reduced tensile strength of the zoite stages in which they are expressed (Khater et al., 2004; Tremp & Dessens, 2011; Tremp et al., 2008; Volkmann et al., 2012). Besides roles in morphogenesis and mechanical strength, the *Plasmodium* alveolins are also involved in gliding motility in both ookinetes and sporozoites, most likely through interactions with components of the glideosome that are situated within the pellicular cytoplasm (Khater et al., 2004; Tremp & Dessens, 2011; Tremp et al., 2008; Volkmann et al., 2012).

In this study, we investigate the expression, subcellular distribution and function of two further members of the alveolin/IMC1 protein family, *Pb*IMC1c and *Pb*IMC1e, revealing fundamental differences in the manner they are expressed and participate in zoite morphogenesis. In addition, we provide the first evidence that both *Pb*IMC1c and *Pb*IMC1e are essential for the development of the asexual blood stages of the parasite in the host, underpinning the alveolins as potential target molecules for chemotherapy-based intervention.

## Materials and methods

### Animal use

All laboratory animal work undergoes regular ethical review by the London School of Hygiene and Tropical Medicine and has been approved by the United Kingdom Home Office. Work was carried out in accordance with the United Kingdom Animals (Scientific Procedures) Act 1986 implementing European Directive 2010/63 for the protection of animals used for experimental purposes. Experiments were conducted in 6–8-week-old female CD1 mice, specific pathogen free and maintained in filter cages. Animal welfare was assessed daily, and animals were humanely killed upon reaching experimental or humane endpoints. Mice were infected with parasites suspended in RPMI or PBS by intraperitoneal injection or by infected mosquito bite on anaesthetized animals. Parasitemia was monitored regularly by collecting a small drop of blood from a superficial tail vein. Drugs were administered by intraperitoneal injection or, where possible, supplied in drinking water. Parasitized blood was harvested by cardiac bleed under general anaesthesia without recovery.

### Parasite maintenance, transmission, culture and purification

*P. berghei* ANKA clone 234 parasites were maintained as cryopreserved stabilates or by mechanical blood passage and regular mosquito transmission. Ookinete cultures were set up overnight from gametocytemic blood as previously described (Arai et al., 2001). After 18–20 h, ookinetes were purified via ice-cold 0.17M ammonium chloride lysis and centrifugation at 800×*g* for 10 min, followed by PBS washes. Mosquito infection and transmission assays were previously described using *Anopheles stephensi* (Dessens et al., 1999; Khater et al., 2004), and infected insects were maintained at 20 °C at approximately 70 % relative humidity.

### Gene-targeting constructs

The entire *pbimc1c* coding sequence plus ca. 0.55 kb of upstream sequence was PCR amplified from *P. berghei* genomic DNA with primers pDNR-IMC1c-F (ACGAAGTTATCAGTCGACGGTACCAAGTGCATTTAGTATGTTGTGGC) and pDNR-IMC1c-R (ATGAGGGCCCCTAAGCTTCTGCATGTACCTGTACAGCAT) and cloned into *Sal*I/*Hin*dIII-digested pDNR-EGFP (Tremp et al., 2008) by in-fusion cloning to give plasmid pDNR-IMC1c/GFP. The 3′UTR of *pbimc1c* was amplified with primers pLP-IMC1c-F (ATATGCTAGAGCGGCCTTTCGTGAAAAATGCAGTTAACA) and pLP-IMC1c-R (CACCGCGGTGGCGGCCGAAAGAAGACAATAAATAAAATAGAAAGTATGG) and the resulting ca. 0.6 kb fragment cloned into *Not*I-digested pLP-hDHFR by in-fusion cloning to give plasmid pLP-hDHFR/IMC1c. The *pbimc1c*/*gfp*-specific sequence from pDNR-IMC1c/GFP was transferred to pLP-hDHFR/IMC1c by Cre/*lox*P recombination to give the final construct pLP-IMC1c/GFP. This plasmid served as template in a PCR-based site-directed mutagenesis using primers IMC1c-KO-F (CAACCGTCATGAGTAAAGGAGAAGAACTTTTCAC) and IMC1c-KO-R (TTACTCATGACGGTTGATGTCTCTTTAGTGT). The resulting PCR product was circularized using in-fusion to give plasmid pLP-IMC1c-KO. In this plasmid, the *pbimc1c* coding sequence except for the first amino acids has been removed.

The entire *pbimc1e* coding sequence plus ca. 0.58 kb of upstream sequence was PCR amplified from genomic DNA with primers pDNR-IMC1e-F (ACGAAGTTATCAGTCGACGGTACCGCATAAATTAACTTAGTTTCATTGAACTTC) and pDNR-IMC1e-R (ATGAGGGCCCCTAAGCTTTCGTTTAAGACGGGTGGTAC) and cloned into *Sal*I/*Hin*dIII-digested pDNR-EGFP by in-fusion cloning to give plasmid pDNR-IMC1e/GFP. The 3′UTR of *pbimc1e* was amplified with primers pLP-IMC1e-F (ATATGCTAGAGCGGCCTTTGGCTTCGATTTTTGTG) and pLP-IMC1e-R (CACCGCGGTGGCGGCCTAACAGCATTATGAAAGATTGGC) and the resulting ca. 0.87 kb fragment cloned into *Not*I-digested pLP-hDHFR by in-fusion cloning to give plasmid pLP-hDHFR/IMC1e. The *pbimc1e*/*gfp*-specific sequence from pDNR-IMC1e/GFP was transferred to pLP-hDHFR/IMC1e by Cre/*lox*P recombination to give the final construct pLP-IMC1e/GFP. This plasmid served as template in a PCR-based site-directed mutagenesis using primers IMC1e-KO-F (AATATGTGATGAGTAAAGGAGAAGAACTTTTCAC) and IMC1e-KO-R (TTACTCATCACATATTTAGTGCCACAATTGC). The resulting PCR product was circularized using in-fusion to give plasmid pLP-IMC1e-KO. In this plasmid, the *pbimc1e* coding sequence except for the first amino acids has been removed.

To generate a mCherry-tagged version in *Pb*IMC1c, the mCherry coding sequence was amplified from pDNR-mCherry/PbSR/EGFP (Carter et al., 2008) with primers pDNR-mCherry-F (CAGTCGACTTAAGCTTAGGGGCCCTCATGGTGAGCAAGGGCG) and pDNR-mCherry-R (AACGGGATCTTCTAGTTACTTGTACAGCTCGTCCATGC) and introduced into *Hin*dIII/*Xba*I-digested pDNR-EGFP by in-fusion to give plasmid pDNR-mCherry. A 3.8-kb fragment corresponding to the entire *pbimc1c* gene plus upstream intergenic region was PCR amplified from *P. berghei* gDNA using primers IMC1c-mCherry-F (ACGAAGTTATCAGTCGAGGTACCTTCTCATTGTCAATGGCTCC) and pDNR-imc1c-R and introduced into *Sal*I/*Hin*dIII-digested pDNR-mCherry by in-fusion to give plasmid pDNR-IMC1c/mCherry. The *Pb*IMC1c/mCherry-specific sequence from pDNR-IMC1c/mCherry was introduced into plasmid pLP-hDHFR/IMC1c by Cre/lox recombination to give plasmid pLP-IMC1c/mCherry/hDHFR.

### Generation and genomic analysis of genetically modified parasites

Parasite transfection, pyrimethamine selection and dilution cloning were performed as previously described (Waters et al., 1997). Prior to performing transfections, plasmid DNA was digested with *Kpn*I and *Sac*II to remove the plasmid backbone. Genomic DNA extraction was performed as previously described (Dessens et al., 1999). For the FP-tagged lines, confirmation of correct targeting and integration into the *pbimc1c* and *pbimc1e* loci was carried out with diagnostic PCR across the integration sites using primer pair hDHFR/ERI-F (ACAAAGAATTCATGGTTGGTTCGCTAAACT) and IMC1c-3′R (TTAGAGCCGATTTTATCTTGTTACAC) for parasite lines IMC1c/GFP and IMC1c/mCherry; and hDHFR/ERI-F and IMC1e-3′R (AAGGTATAAAGTTTATGCATTTTAGCTATC) for parasite line IMC1e/GFP. Confirmation of the absence of the WT allele in the transgenic lines was carried out with primer pairs pDNR-IMC1c-F and IMC1c-3′R (for IMC1c/GFP); IMC1c-5′F (CTATACCACGCAGCAACAATG) and IMC1c-3′R (for IMC1c/mCherry); and pDNR-IMC1e-F and IMC1e-3′R (for IMC1e/GFP).

### Western blot analysis

Parasite samples were heated directly in SDS-PAGE loading buffer at 70 °C for 10 min. Proteins were fractionated by electrophoresis through NuPage 4–12 % Bis-Tris precast gels (Invitrogen) and transferred to PVDF membrane (Invitrogen) according to the manufacturer’s instructions. Membranes were blocked for non-specific binding in PBS supplemented with 0.1 % Tween 20 and 5 % skimmed milk for 1 h at room temperature. Goat polyclonal antibody to green fluorescent protein (GFP) conjugated to horse radish peroxidase (Abcam ab6663) diluted 1:5,000 was applied to the membrane for 1 h at room temperature. After washing, signal was detected by chemiluminescence (Pierce ECL western blotting substrate) according to the manufacturer’s instructions.

### Microscopy

For assessment of fluorescence, live parasite samples were assessed, and images captured, on a Zeiss LSM510 inverted confocal microscope or on a Zeiss Axioplan-2 fluorescent microscope with Retiga 2000R CCD camera system and Volocity software.

## Results

### Structure of the *Plasmodium* alveolins IMC1c and IMC1e

**Fig. 1:**
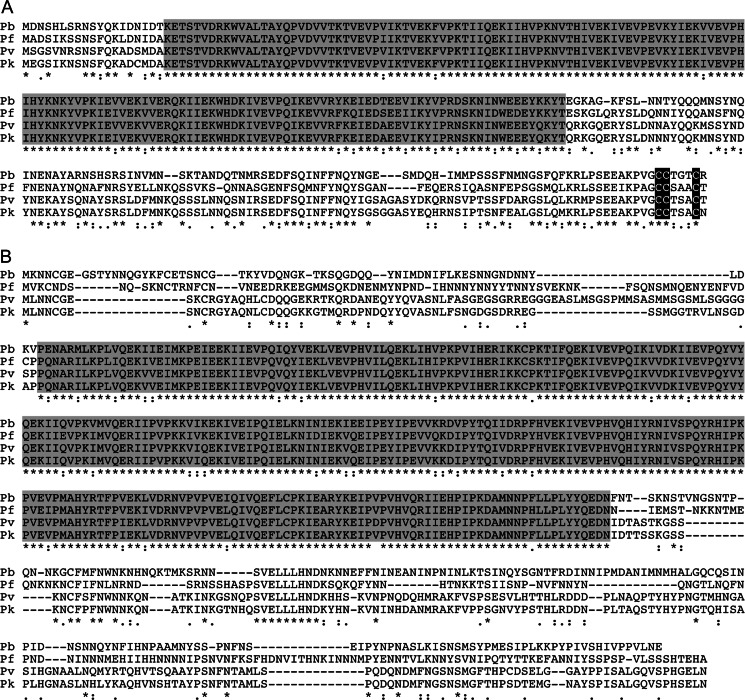
Sequence and structure of *Pb*IMC1 proteins. Multiple amino acid sequence alignment of the predicted IMC1c (**a**) and IMC1e (**b**) proteins from *P. berghei* (Pb), *P. knowlesi* (Pk), *P. vivax* (Pv) and *P. falciparum* (Pf). Indicated are conserved alveolin domains (*shaded*) and gaps introduced to allow optimal alignment (*hyphens*). Conserved amino acid identities (*asterisks*) and similarities (*colons* and *points*) are indicated underneath. Also shown is a conserved cysteine motif at the carboxy-terminus of IMC1c (*black shading*). The alignment was made with ClustalW

The *Plasmodium* IMC1 protein family was first published in 2004 using gene models of *P. yoelii* (Khater et al., 2004). *Pb*IMC1c (PBANKA_120200) is composed of 278 amino acids encoded by a single exon. *Pb*IMC1c and its orthologous proteins share a highly conserved amino-terminal domain related to the IMCp domain superfamily (Pfam12314) that defines the IMC1 proteins/alveolins (Fig. 1a). The proteins also possess a conserved cysteine motif at the carboxy-terminus similar to the cysteine motifs described in *Pb*IMC1a and *Tg*IMC1 (Fig. 1a) that is believed to act as a palmitoylation signal (Khater et al., 2004; Mann & Beckers, 2001). *Pb*IMC1e (PBANKA_040270) is composed of 512 amino acids encoded by a single exon. Sequence conservation is limited to an IMCp domain in their central portions (Fig. 1b). Interestingly, the *Plasmodium imc1e* locus is located directly downstream of its family member *imc1a* in the opposite orientation, suggesting that these two genes could be sharing promoter elements.

### Fluorescent protein tagging of PbIMC1c and PbIMC1e

**Fig. 2:**
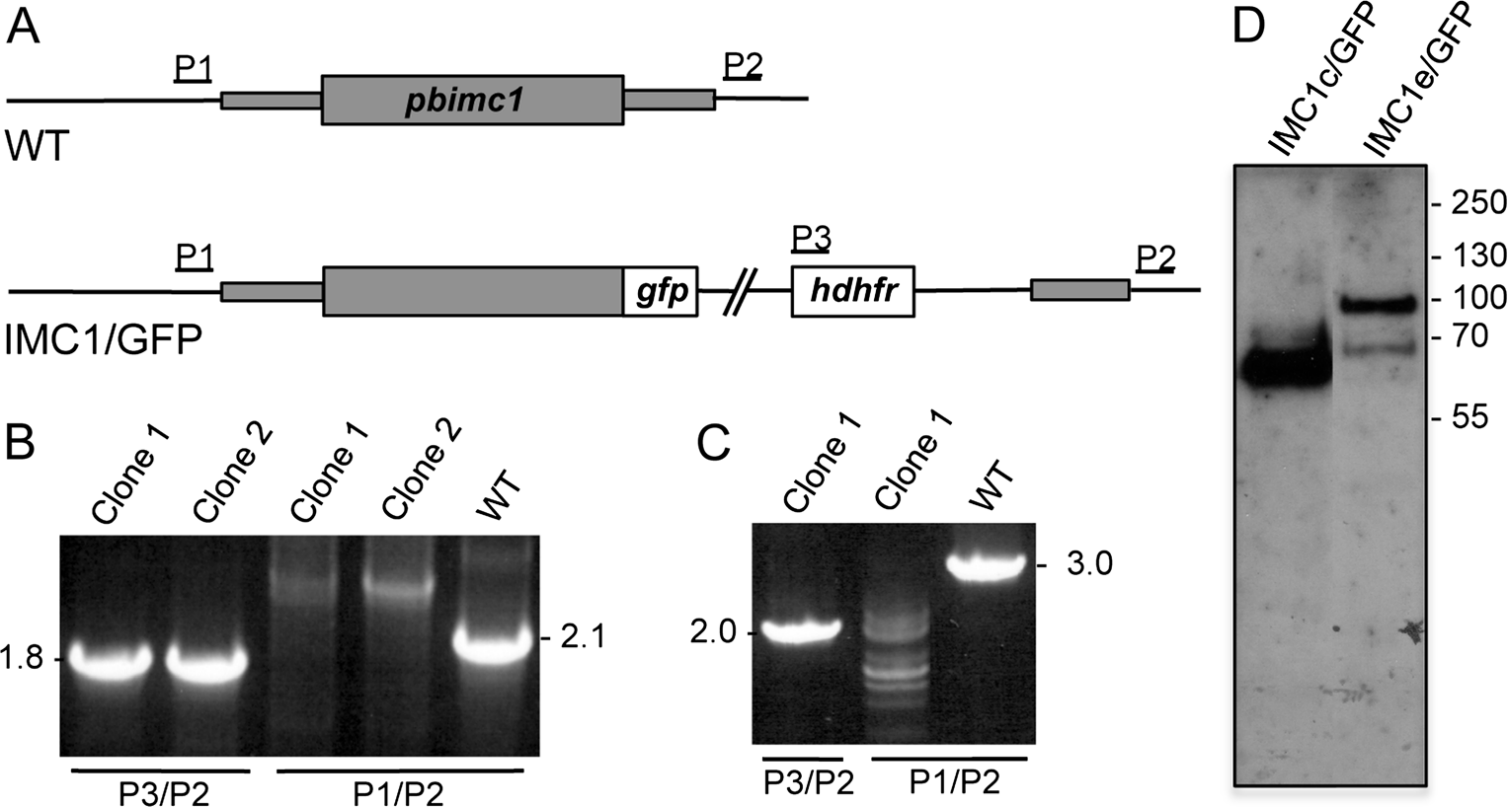
Generation and molecular analyses of genetically modified parasite lines. **a** General targeting strategy for the GFP tagging of *pbimc1c* and *pbimc1e* via double crossover homologous recombination. Both the wild-type (WT) and modified, GFP-tagged (IMC1/GFP) alleles are shown. The *pbimc1* gene is indicated with coding sequence (*wide bars*) and non-coding sequence (*narrow bars*). Also indicated are the enhanced GFP module (*gfp*), the hDHFR selectable marker gene cassette (*hdhfr*) and primers used for diagnostic PCR amplification (P1-P3). **b** PCR diagnostic for the presence of the GFP-tagged *pbimc1* alleles using primers P2 and P3 (P3/P2) and the absence of the wild-type *pbimc1* alleles using primers P1 and P2 (P1/P2) from clonal parasite populations of IMC1c/GFP (*left panel*) and IMC1e/GFP (*right panel*). WT parasites are included as positive controls for the unmodified alleles. **c** Western blot analysis of purified, cultured ookinete samples of parasite lines IMC1c/GFP and IMC1e/GFP using anti-GFP antibodies, showing corresponding *Pb*IMC1::GFP fusion proteins

To achieve GFP tagging of *Pb*IMC1c and *Pb*IMC1e, we adopted a strategy of double crossover homologous recombination in which the wild-type alleles were replaced with recombinant full-length wild-type alleles fused to GFP at their carboxy-terminus (Fig. 2a). After the transfection of purified schizonts, pyrimethamine-resistant parasites were selected and cloned by limiting dilution as described (Tremp & Dessens, 2011; Tremp et al., 2008) to give parasite lines IMC1c/GFP and IMC1e/GFP, respectively. PCR diagnostic for integration into the *pbimc1c* locus produced a specific band of 1.8 kb in the IMC1c/GFP clones, while PCR diagnostic for the presence of the wild-type *imc1c* allele gave a specific band of 2.1 kb only in wild-type parasites (Fig. 2b). Likewise, PCR diagnostic for integration into the *pbimc1e* locus produced a specific band of 2.0 kb in the IMC1e/GFP clones, while PCR diagnostic for the presence of the wild-type *pbimc1e* allele gave a specific band of 3.0 kb only in wild-type parasites (Fig. 2b). Both genetically modified parasite lines generated displayed normal parasite development in mouse and mosquito and were readily transmitted by sporozoite-infected mosquito bites, indicating that the carboxy-terminal GFP fusions had not adversely affected the function of *Pb*IMC1c and *Pb*IMC1e. Both parasite lines displayed GFP fluorescence in ookinetes (see below), and immuno blot analysis of purified, cultured ookinetes with anti-GFP antibodies detected specific bands corresponding to the *Pb*IMC1c and *Pb*IMC1e fusion proteins with GFP, respectively (Fig. 2c).

### Life-stage expression of PbIMC1c and PbIMC1e

**Fig. 3:**
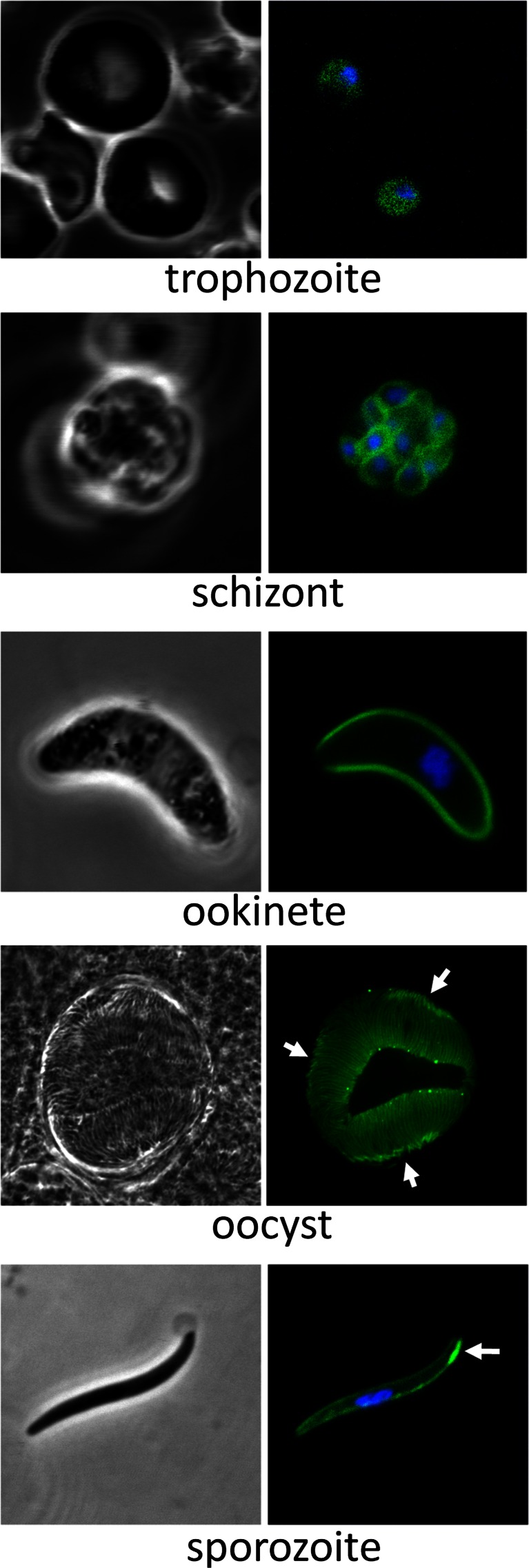
Expression and subcellular localization of *Pb*IMC1c. Confocal bright-field and GFP fluorescence images of trophozoite, schizont, ookinete, mature oocyst and sporozoite life stages. Hoechst DNA staining (*blue*) indicates position of nuclei. *Arrows* point to anterior structures in sporozoites

The expression and subcellular distribution of *Pb*IMC1c and *Pb*IMC1e were assessed by UV and laser scanning microscopy of live parasites. IMC1c/GFP parasites displayed strong fluorescence throughout asexual blood-stage development that appeared cytoplasmic, except in mature schizonts where it showed clear peripheral localization in individual merozoites (Fig. 3). To assess *Pb*IMC1c expression in the mosquito stages, we set up ookinete cultures and infected *A. stephensi* vector mosquitoes. Cultured ookinetes displayed very strong fluorescence with a cortical distribution (Fig. 3). Sporulated oocysts and sporozoites also displayed strong fluorescence, which was concentrated at the cortex of the sporozoites (Fig. 3). These combined observations are fully consistent with a pellicular localization of *Pb*IMC1c and are in agreement with it being a predicted SPN resident. Besides the peripheral distribution of *Pb*IMC1c in sporozoites, a thickened area was present near one extremity of the cell (Fig. 3). In *P. berghei* sporozoites, the nucleus is consistently positioned closer to the posterior end of the cell (Kudryashev et al., 2010). Accordingly, based on its position relative to the sporozoite nucleus, as well as its localization away from the sporoblast in sporulated oocysts (Fig. 3), the discrete area of fluorescence appears to be located at the anterior end.

**Fig. 4:**
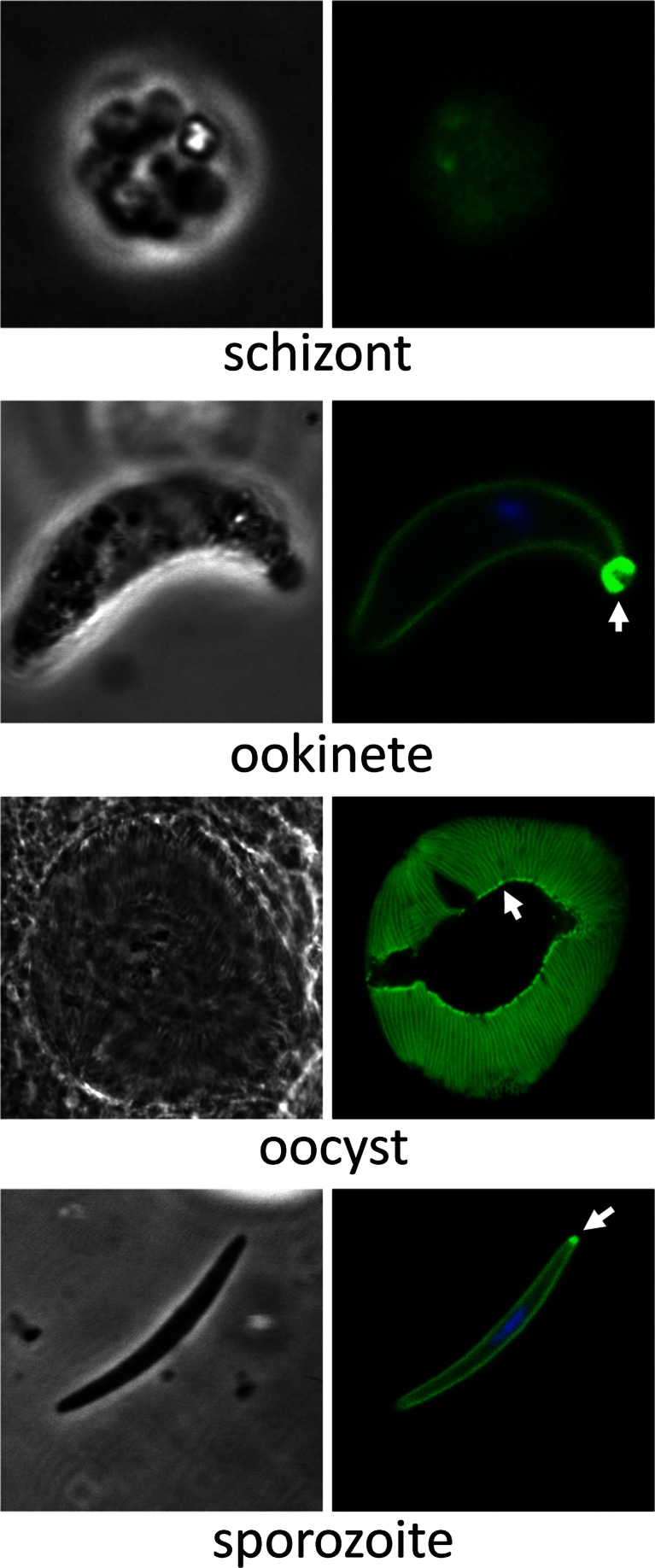
Expression and subcellular localization of *Pb*IMC1e. Bright-field and GFP fluorescence images of schizont, ookinete, mature oocyst and sporozoite life stages. The schizont image was captured using a CCD camera due to the low levels of fluorescence, while the other images were captured by confocal microscopy. Hoechst DNA staining (*blue*) indicates the position of nuclei. *Arrows* point to posterior structures in ookinetes and sporozoites

In contrast to IMC1c/GFP parasites, IMC1e/GFP parasites exhibited very weak GFP-based fluorescence in blood stages that required recording with a CCD digital microscope camera (Fig. 4). Because of these low fluorescence levels, it was difficult to discern a specific subcellular distribution. In contrast to the blood stages, mature ookinetes displayed much stronger GFP fluorescence that was distributed predominantly in the cell cortex (Fig. 4), consistent with a pellicular localization of the protein and its predicted function in the SPN. *Pb*IMC1e was also present in an unknown structure situated at one extremity of the ookinete (Fig. 4). A genetic cross with parasite line G2/GFP, which labels the collar (i.e. an apical cap-like structure of the ookinete) (Tremp et al., 2013), gave rise to heterokaryotic ookinetes that simultaneously displayed both the *Pb*G2-labelled collar and *Pb*IMC1e-labelled structure (data not shown), indicating that the latter is positioned at the posterior end of the ookinete. Sporulated oocysts and sporozoites also displayed GFP florescence, which localized to the periphery of the sporozoites (Fig. 4). Sporozoites possessed a small fluorescent spot at one extremity which, based on its position relative to the sporozoites nucleus, as well as its localization in sporulated oocysts lining the sporoblast (Fig. 4), appears to correspond to the sporozoite posterior end.

### PbIMC1c and PbIMC1e display distinct temporal recruitment to the SPN

**Fig. 5:**
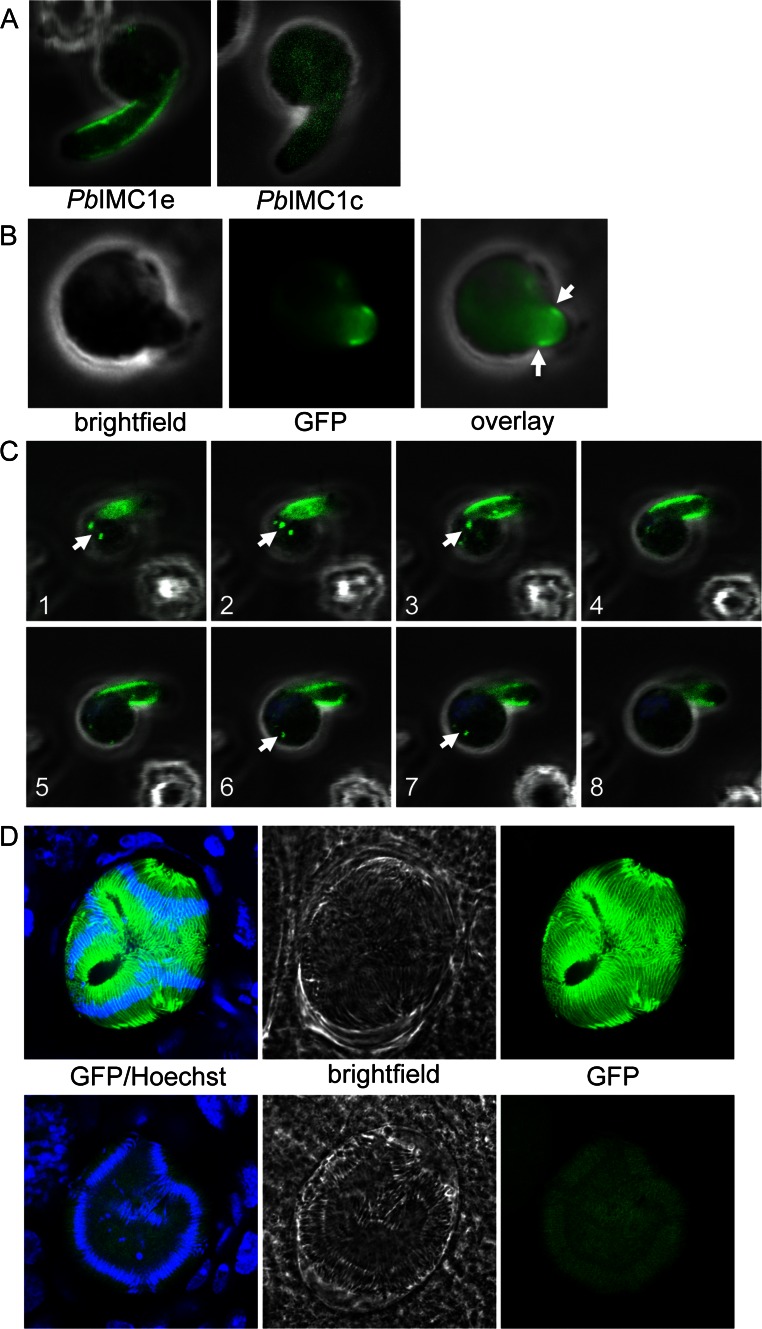
Recruitment of *Pb*IMC1 proteins to the pellicle. **a** Retort stages at approximately 6 h post-gametogenesis showing the presence (*Pb*IMC1e) and absence (*Pb*IMC1c) of pellicular localization. **b** Very young retort of parasite line IMC1e/GFP at approximately 4 h post-gametogenesis, exhibiting pellicular localization (*arrows*). **c** Serial Z-stack images of a young retort of parasite line IMC1e/GFP, exhibiting fluorescent spots within the spherical part (*arrows*). **d** Confocal bright-field and GFP fluorescence images of sporulated oocysts of parasite line IMC1c/GFP, exhibiting strong peripheral fluorescence in the sporozoites (*top panels*) or very weak cytoplasmic fluorescence (*bottom panels*). The GFP fluorescence image in the bottom panel was captured using increased photomultiplier gain. The *top left hand corner* shows part of a not yet sporulated oocyst containing multiple round nuclei (*blue*) and low-level GFP fluorescence

To further study the recruitment of *Pb*IMC1c and *Pb*IMC1e to the pellicle, we examined retorts (i.e. immature ookinetes), which contain an elongated ‘ookinete’ portion that contains pellicle and a spherical ‘zygote’ part that does not. This revealed a marked difference between the two alveolins: whereas *Pb*IMC1e was clearly localized to the periphery of the elongated ‘ookinete’ portion of the retort, *Pb*IMC1c was not (Fig. 5a). In fact, *Pb*IMC1e was detected in the pellicle of very young retorts, indicating that it is assembled into the ookinete SPN from the start of pellicle/SPN formation (Fig. 5b). Interestingly, this process was accompanied by the formation of several fluorescent spots in the spherical ‘zygote’ section (Fig. 5c).

When examining oocysts on IMC1c/GFP parasite-infected *A. stephensi* midguts at 2 weeks post-infection, we observed fully sporulated oocysts with very strong GFP fluorescence that clearly was localized at the sporozoites’ cortex (Fig. 5d). However, on the same midguts, we found sporozoite-containing oocysts that exhibited very low, baseline GFP fluorescence levels similar to not yet sporulated oocysts (Fig. 5d). These observations indicate that *Pb*IMC1c is predominantly expressed after sporozoite budding and—similar to the situation in the ookinete—is recruited to the SPN after pellicle formation. By contrast, IMC1e/GFP parasite-infected midguts had sporulated oocysts that exhibited strong fluorescence without exception.

### PbIMC1c and PbIMC1e are differentially expressed from maternal and paternal alleles in the sexual stages

**Fig. 6:**
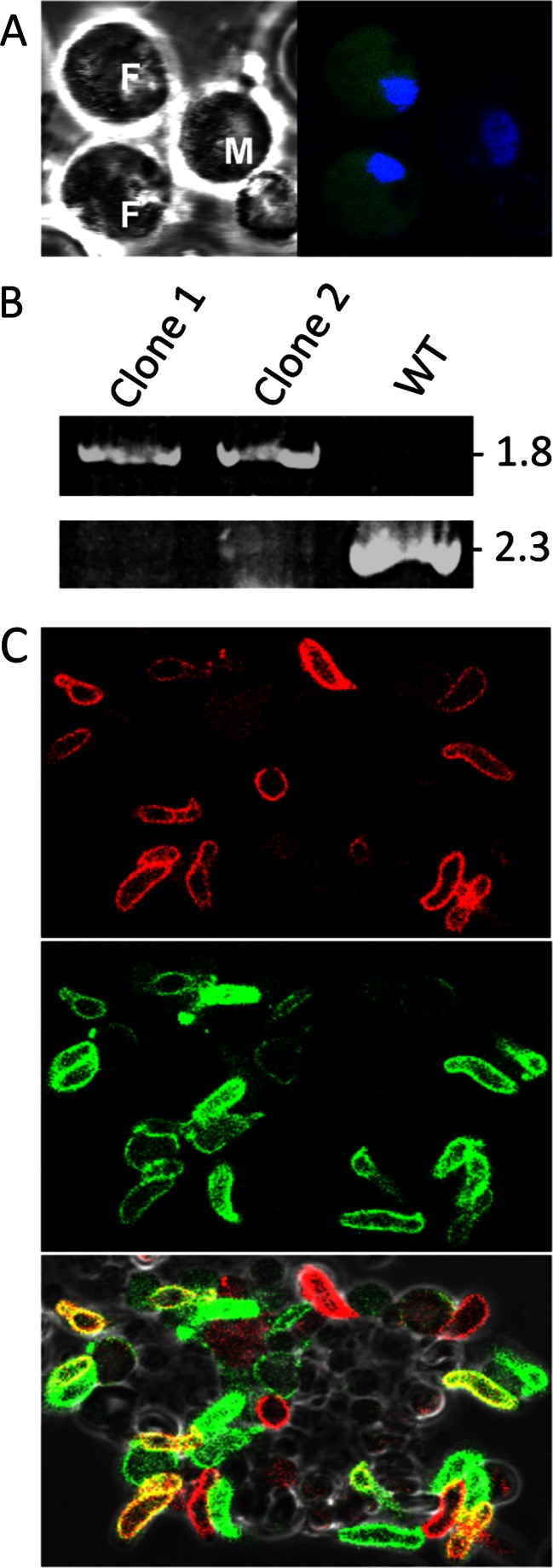
Expression of *Pb*IMC1c from parental alleles in the sexual stages. **a** Confocal bright-field and GFP fluorescence images of female (*F*) and male (*M*) gametocytes of parasite line IMC1c/GFP. Hoechst staining (*blue*) labels nuclei. **b** PCR diagnostic for the integration of the selectable marker into the *pbimc1c* locus in two different clones of parasite line IMC1c/mCherry, amplying a 1.8-kb fragment (*top panel*). PCR diagnostic for the unmodified *pbimc1c* allele amplified a 2.3-kb product only from WT parasites (*bottom panel*). **c** Confocal images of ookinetes derived from a genetic cross between parasite lines IMC1c/GFP and IMC1c/mCherry, showing mCherry (*top*) and GFP fluorescence (*middle*). *Bottom panel* shows overlay with bright field and identifies dual-labelled ookinetes (*yellow*) pointing to expression from both parental alleles

Interestingly, we found a weak cytoplasmic *Pb*IMC1c::GFP expression in gametocytes, but only in females (possessing the smaller nucleus) (Fig. 6a). We did not see a discernible increase in fluorescence until some 7–8 h post-gametogenesis, resulting in mature ookinetes at 24-h ookinetes with very strong fluorescence levels (Fig. 3). To test IMC1c expression from the paternal allele, parasite line IMC1c/mCherry was generated to express a red fluorescent protein-tagged version of *Pb*IMC1c, which was then crossed with the equivalent GFP-tagged parasite line. The strategy used to generate IMC1/mCherry was the same as for IMC1c/GFP (Fig. 2). Accordingly, PCR diagnostic for the integration of the selectable marker into the *pbimc1c* locus amplified a 1.8-kb fragment from different clones of this parasite line and not from wild-type parasites, as expected (Fig. 6b). Additionally, PCR diagnostic for the wild-type *pbimc1c* allele amplified a 2.3-kb product from wild-type parasites, but not from the transgenic lines, as expected (Fig. 6b). The resulting *Pb*IMC1c::mCherry fusion protein displayed similar life-stage expression and subcellular distribution as its GFP-tagged counterpart (data not shown and Fig. 6c). After a genetic cross with parasite line IMC1c/GFP, heterozygous ookinetes (derived from cross fertilization) were produced that dually expressed red and green fluorescent proteins (Fig. 6c). This demonstrates that *Pb*IMC1c is expressed from both the maternal- and paternal-inherited alleles in the mature ookinete. In a time course, dual expression of GFP and mCherry was first detected at approximately 7 h post-gametogenesis, indicating this is the point when protein expression from the paternal *pbimc1c* allele commences.

Parasite line IMC1e/GFP exhibited very weak GFP fluorescence in gametocytes (data not shown). In this parasite, fluorescence levels increased around 4 h post-gametogenesis prior to the start of pellicle formation. To test *Pb*IMC1e expression from the paternal allele, we crossed parasite line IMC1e/GFP with parasite line PbSR/EGFP (Carter et al., 2008). The latter expresses a GFP-tagged version of *Pb*LAP1, which is maternally inherited (Raine et al., 2007). In mature ookinetes, *Pb*LAP1 is almost exclusively present in the crystalloids, which appear as one or two distinctive fluorescent spots in ookinetes of parasite line *Pb*SR/EGFP (Carter et al., 2008). In the crossed ookinete culture, we could not detect any mature heterokaryotic ookinetes that displayed, at the same time, fluorescent crystalloids and a fluorescent cortex (64 % only peripheral GFP, 36 % only crystalloid GFP; *n* = 100). A similar result (59 % only peripheral GFP, 41 % only crystalloid GFP; *n* = 100) was obtained when we crossed IMC1e/GFP with parasite line *Pb*LAP3/GFP, which expresses a GFP-tagged family member of *Pb*LAP1 that is also maternally expressed (Saeed et al., 2010, 2012). These observations show that, in contrast to *Pb*IMC1c, *Pb*IMC1e is only expressed from the maternal allele in the sexual stages.

### PbIMC1c and PbIMC1e are essential for blood-stage asexual parasite development

**Fig. 7:**
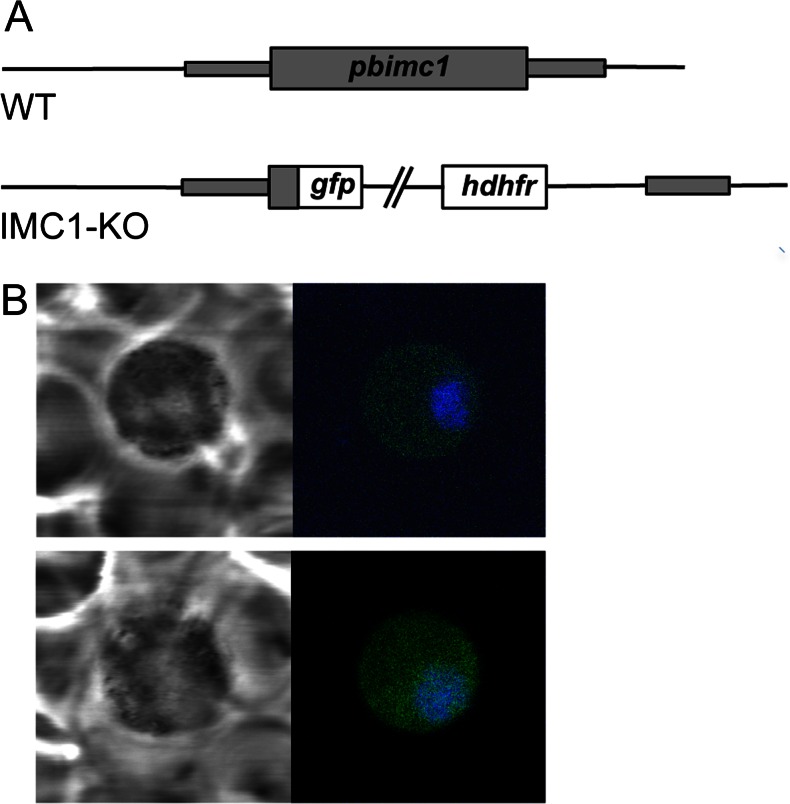
Targeted disruption of *Pb*IMC1c and *Pb*IMC1e. **a** Gene structure of *pbimc1* alleles in WT and *Pb*IMC1-KO *parasite lines*. The *pbimc1* gene is indicated with coding sequence (*wide bars*) and non-coding sequence (*narrow bars*). Also indicated are the enhanced GFP module (*gfp*) and the hDHFR selectable marker gene cassette (*hdhfr*). **b** Confocal bright-field and GFP fluorescence images of gametocytes after attempted disruption of *pbimc1c* (*top panel*) and *pbimc1e* (*bottom panel*). Hoechst DNA staining (*blue*) labels nuclei

To achieve knockout of *Pb*IMC1c and *Pb*IMC1e expression, we again adopted a strategy of double crossover homologous recombination identical to the GFP-tagging approach. The coding sequences of *pbimc1c* and *pbimc1e* were removed leaving GFP under control of the native *pbimc1* gene promoters to act as a reporter (Fig. 7a). In contrast to the transfections aimed at GFP tagging, which readily resulted in a specific integration into the *pbimc1c* and *pbimc1e* loci (Fig. 2), our attempts to disrupt *pbimc1c* and *pbimc1e* repeatedly failed to give integration of the selectable marker into the target loci (based on five independent transfections for each gene knockout). This indicated that these genes are important for the development of asexual blood stages and cannot be disrupted, which is consistent with the observed expression of these genes in asexual blood-stage parasites (Figs. 3 and 4). This notion was corroborated by the fact that the ‘knockout’ transfections produced drug-resistant parasites that displayed green fluorescence resulting from the expression of the GFP reporter gene. However, in both cases, the GFP fluorescence was only observed in gametocytes, with fluorescence levels being markedly stronger after targeting *pbimc1e* than after targeting *pbimc1c* (Fig. 7b). The lack of integration of the selectable marker and GFP reporter into the target loci, combined with the clear disparity between the expression profiles of GFP in these transfections compared to those that generated GFP-tagged *Pb*IMC1c and *Pb*IMC1e (Figs. 3 and 4), strongly points to integration into non-specific genomic locations. Such events are likely to be selected only when homologous recombination is detrimental to parasite development. Hence, these observations strongly support a critical role for *Pb*IMC1c and *Pb*IMC1e in asexual blood-stage development of the parasite.

## Discussion

This study shows that a further two members of the *Plasmodium* alveolin/IMC1 protein family are recruited to the SPN in the zoite stages where they are expressed as protein. This faithful localization to the pellicle further supports the notion that alveolins have a predominantly cytoskeletal function and, hence, that the structural similarities (i.e. the IMCp domains) reflect functional properties. We show that *Pb*IMC1c and *Pb*IMC1e are expressed in all three zoite stages of the malaria parasite including merozoites. Recent studies based on cryo-electron tomography failed to detect an apparent subpellicular structure in merozoites, suggesting that a SPN may not be present in this zoite stage (Kudryashev et al., 2012). However, the clear peripheral distribution of *Pb*IMC1c in merozoites shown here (Fig. 3) supports the presence of a SPN within the merozoite pellicle. The IMC1 protein expression profiles thus far established by us and others in *P. berghei* (Khater et al., 2004; Kono et al., 2012; Tremp & Dessens, 2011; Tremp et al., 2008; Volkmann et al., 2012) fit very well with available *Plasmodium falciparum* protein expression data (Florens et al., 2002; Hall et al., 2005; Lasonder et al., 2002; Lindner et al., 2013; Treeck et al., 2011), indicating that *Plasmodium* alveolin orthologues have conserved stage-specific expression profiles. The new alveolin expression data reported here are therefore likely to apply also to *P. falciparum* and other human malaria species. An exception to this may be the gametocyte, which possesses a pellicle structure in *P. falciparum*, but not in *P. berghei* (Dearnley et al., 2012). Moreover, antibodies to a generic alveolin epitope label the periphery of *P. falciparum* gametocytes (Gould et al., 2008), indicating that alveolins are indeed present in the gametocyte SPN.

Our data show for the first time that clear differences exist between *Plasmodium* alveolins with respect to their assembly into the SPN of ookinetes and sporozoites (Fig. 5). Whereas *Pb*IMC1e appears to be assembled concurrent with pellicle formation, *Pb*IMC1c joins the SPN only after pellicle formation. Accordingly, we anticipate that *Pb*IMC1c is not required for normal ookinete and sporozoite morphogenesis, in contrast to its family members *Pb*IMC1a, *Pb*IMC1b and *Pb*IMC1h (Khater et al., 2004; Tremp & Dessens, 2011; Tremp et al., 2008). Our observations provide a clear demonstration that the SPN continues to develop after zoite formation. This could, for instance, explain why cryo-electron tomography points to midgut sporozoites having a less prominent SPN than salivary gland sporozoites (Kudryashev et al., 2012). Our observations are also consistent with studies of *Toxoplasma* showing that the SPN of older parasites becomes detergent insoluble, reflecting a change in rigidity and mechanical strength of the structure (Mann et al., 2002). These observations all point to a process of maturation of the SPN after its initial biogenesis.

There are clear parallels between the *Plasmodium* alveolins described here and some of those characterized in *Toxoplasma gondii* (Anderson-White et al., 2011). For example, *T. gondii* IMC1, IMC3, IMC6 and IMC10 localize to the cortical cytoskeleton during tachyzoite daughter cell budding, whereas IMC7, IMC12 and IMC14 are only found in the mature pellicles and not in those of the emerging daughter cells (Anderson-White et al., 2011). Even though the alveolin repertoires differ between *Plasmodium* and *Toxoplasma* (Anderson-White et al., 2011; Kono et al., 2012), the distinct chronological assembly of certain family members into the SPN appears to be a common feature that is likely to reflect a biological requirement for different physical properties of the SPN at different phases of zoite development. Another similarity with *Toxoplasma* alveolins is that, although their main site of action is the cortical cytoskeleton, some are also found in additional subcellular structures such as the basal body and centrosome (Anderson-White et al., 2011). *Pb*IMC1c and *Pb*IMC1e, too, localize to additional structures (Figs. 3 and 4). For the ookinete, defined basal structures that could correspond to the posterior structure containing *Pb*IMC1e have not been described. It is notable that the posterior structure associated with the ookinete appears almost exterior of the cell, indicating that it could constitute residual ‘zygote’ material left over from the transformation of the spherical zygote into the elongated ookinete. Notably, assembly of *Pb*IMC1e into the pellicle is accompanied by the formation of multiple discrete *Pb*IMC1e-containing ‘spots’ that lie mainly within the cytoplasm of the spherical zygote portion, which may become trapped within the residual zygote membrane at the posterior end of the cell. Similar spots were not apparent during the formation of ookinetes that express GFP-tagged *Pb*IMC1b or *Pb*IMC1h (Tremp & Dessens, 2011; Tremp et al., 2008). In sporozoites, the posterior structure that is labelled with *Pb*IMC1e could correspond to, or co-localize with, the posterior polar ring (Kudryashev et al., 2010). It is also not clear what the apical structure in sporozoites labelled with *Pb*IMC1c corresponds to. It is notable that the area is present only on one side of the anterior sporozoite, and one possibility is that it could co-localize with the apical ring complex that sits at a sharp angle towards the ventral side of the sporozoite tip (Kudryashev et al., 2012).

Although *Pb*IMC1c is present in asexual, sexual and sporogonic life stages, it is not constitutively expressed as the protein was not detected in male gametocytes or in oocysts before sporulation. The apparent lack of GFP fluorescence in male gametocytes indicates that *Pb*IMC1c is not carried over from the preceding trophozoite stage; if this was the case, both male and female gametocytes would be expected to express GFP. Rather, the restricted expression in female gametocytes points to an early commitment to sexual stage development that occurs before trophozoite development. Female gametocytes in *P. berghei* are spherical cells that do not possess a discernible pellicle, so it is not clear why the protein is expressed here. One possibility is that *Pb*IMC1c could have a function in the gametocyte that is not linked to the SPN. It should be noted that a study by Mair and colleagues (Mair et al., 2006) shows significantly reduced transcript levels of both *pbimc1c* and *pb1mc1e* in gametocytes of the helicase DOZI (development of zygote inhibited) null mutant parasites. This suggests that these genes are subject to translational repression, a female gametocyte-specific mechanism of translational silencing involved in the development of the parasite post-fertilization (Mair et al., 2006). Translational repression of *pbimc1c* and *pbimc1e* is consistent with the failure to detect significant amounts of the respective gene products in gametocytes using high-accuracy mass spectrometry-based proteomics (Hall et al., 2005; Khan et al., 2005). In *P. falciparum*, too, *pfimc1c* and *pfimc1e* mRNAs are abundant in mature blood-stage gametocytes (Lopez-Barragan et al., 2011), while the corresponding gene products have not been detected in this life stage by mass spectrometry (Silvestrini et al., 2010), again supporting a scenario of translational repression. The low expression of *Pb*IMC1c observed in female gametocytes could be the result of ‘leaky’ translational repression, where only a fraction of the *pbimc1c* mRNA is silenced.

The failure to achieve a structural disruption of the *pbimc1c* and *pbimc1e* genes indicates that these genes are refractory to genetic depletion. This, in turn, indicates that these genes are essential for the completion of the cycle of blood-stage schizogony or for infectivity of the merozoites. Besides repeated failure of the transfections aimed at gene ‘knockout’ in contrast to those aimed at gene ‘tagging’, we obtained additional evidence which strongly supports a vital role of *Pb*IMC1c and *Pb*IMC1e in blood-stage parasite development: In both cases the transfections aimed at gene disruption resulted in a non-specific integration of the GFP reporter and the accompanying drug selection marker, giving rise to green fluorescent gametocytes (Fig. 7b). We presume that these events must have occurred via non-homologous recombination-based integration into a ‘random’ gene, leaving its respective promoter to drive GFP reporter expression. Because homologous recombination is much more efficient than non-homologous recombination, the latter is likely to be selected only when homologous recombination is detrimental to parasite development. Interestingly, we obtained similar GFP expression after replicate transfections, suggesting that the integration site may not be entirely indiscriminate and perhaps could constitute the ‘next best’ site with regards to sequence homology with the target DNA. We propose that this phenomenon can be a useful marker for the identification of genes that are vital for asexual development.
